# Cyclooxygenase activity mediates colorectal cancer cell resistance to the omega-3 polyunsaturated fatty acid eicosapentaenoic acid

**DOI:** 10.1007/s00280-020-04157-2

**Published:** 2020-10-11

**Authors:** Milene Volpato, Nicola Ingram, Sarah L Perry, Jade Spencer, Amanda D Race, Catriona Marshall, John M Hutchinson, Anna Nicolaou, Paul M Loadman, P Louise Coletta, Mark A Hull

**Affiliations:** 1 Leeds Institute of Medical Research at St James’s, University of Leeds, St James’s University Hospital, Leeds, LS9 7TF UK; 2 Institute of Cancer Therapeutics, University of Bradford, Bradford, BD7 1DP UK; 3 Laboratory for Lipidomics and Lipid Biology, Division of Pharmacy and Optometry, School of Health Sciences, University of Manchester, Manchester, M13 9PT UK; 4 Lydia Becker Institute of Immunology and Inflammation, University of Manchester, Manchester, M13 9PT UK

**Keywords:** Aspirin, Cancer pharmacology, Celecoxib, Colorectal cancer, Cyclooxygenase, Drug metabolism, Eicosapentaenoic acid

## Abstract

**Purpose:**

The naturally-occurring omega-3 polyunsaturated fatty acid eicosapentaenoic acid (EPA) is safe, well-tolerated and inexpensive, making it an attractive anti-cancer intervention. However, EPA has only modest anti-colorectal cancer (CRC) activity, when used alone. Both cyclooxygenase (COX) isoforms metabolise EPA and are over-expressed in CRC cells. We investigated whether COX inhibition increases the sensitivity of CRC cells to growth inhibition by EPA.

**Methods:**

A panel of 18 human and mouse CRC cell lines was used to characterize the differential sensitivity of CRC cells to the growth inhibitory effects of EPA. The effect of CRISPR-Cas9 genetic deletion and pharmacological inhibition of COX-1 and COX-2 on the anti-cancer activity of EPA was determined using in vitro and in vivo models.

**Results:**

Genetic ablation of both COX isoforms increased sensitivity of CT26 mouse CRC cells to growth inhibition by EPA in vitro and in vivo. The non-selective COX inhibitor aspirin and the selective COX-2 inhibitor celecoxib increased sensitivity of several human and mouse CRC cell lines to EPA in vitro. However, in a MC38 mouse CRC cell tumour model, with dosing that mirrored low-dose aspirin use in humans, thereby producing significant platelet COX-1 inhibition, there was ineffective intra-tumoral COX-2 inhibition by aspirin and no effect on EPA sensitivity of MC38 cell tumours.

**Conclusion:**

Cyclooxygenase inhibition by non-steroidal anti-inflammatory drugs represents a therapeutic opportunity to augment the modest anti-CRC activity of EPA. However, intra-tumoral COX inhibition is likely to be critical for this drug-nutrient interaction and careful tissue pharmacodynamic profiling is required in subsequent pre-clinical and human studies.

**Electronic supplementary material:**

The online version of this article (10.1007/s00280-020-04157-2) contains supplementary material, which is available to authorized users.

## Introduction

Omega-3 polyunsaturated fatty acid (O3FA) supplements containing eicosapentaenoic acid (EPA) and docosahexaenoic acid (DHA) are safe, well-tolerated, with beneficial cardiovascular effects [1, 2] and anti-inflammatory properties [3]. Pure EPA is licensed for use in severe hypertriglyceridemia and has been shown to reduce major cardiovascular and cerebrovascular events in high-risk individuals [4]. Oral EPA [2 g free fatty acid (FFA) daily] has modest primary colorectal cancer (CRC) chemoprevention activity in familial adenomatous polyposis (FAP) and in individuals with ‘sporadic’ colorectal adenomas [5, 6]. O3FAs also have the potential for the treatment of CRC [7]. We have previously shown that EPA inhibits the growth of syngeneic CT26 mouse CRC cell liver tumours in an intra-splenic injection model of CRC liver metastasis (LM) in *BALB/c* mice [8], at levels of tumour EPA incorporation attained in randomised controlled trials (RCTs) [5, 6]. A phase 2 RCT of EPA 2 g FFA daily in patients awaiting liver resection surgery for CRCLM (the EMT trial) showed that EPA was safe and well-tolerated in advanced CRC patients [9]. The EMT trial suggested possible overall survival benefit from EPA for 12–18 months after surgery [9] and has led to the phase 3 EMT2 RCT of long-term treatment with EPA in patients undergoing CRCLM surgery (NCT03428477).

Human CRC cells display differential sensitivity to EPA in vitro. In some cell lines, 50 µM EPA or below is sufficient to have anti-proliferative effects [9–12]. Yet, 150 µM EPA or above is necessary to induce significant growth inhibition in others [13]. Differential sensitivity to EPA is also observed in other cancer cell types with 5–10 µM EPA reported to inhibit the growth of three human pancreatic cancer cell lines [14].

Several mechanisms of action of the anti-CRC activity of EPA have been proposed, including lipid raft modulation and altered metabolism by cyclooxygenase (COX)-1 and -2, lipoxygenases and CYP450 monooxygenases [14–17].

We exploited differential sensitivity of CRC cells to EPA to delineate factors controlling resistance to EPA, with a view to optimising anti-cancer treatment with this O3FA. We identified the COX isoforms as mediators of CRC cell resistance and tested the hypothesis that COX inhibition, which is a clinically relevant intervention using available non-steroidal anti-inflammatory drugs (NSAIDs), increases CRC cell sensitivity to the growth inhibitory effects of EPA.

## Methods

### Materials

Pure EPA-FFA, a gift from SLA Pharma (UK) was used for in vitro experiments [18]. For animal diets, we used 90% EPA-triglyceride (TG) oil (Ingennus Healthcare Nutrition, UK) [6]. Aspirin and celecoxib were purchased from Sigma-Aldrich (UK). A working stock solution of aspirin (50 mg/ml) and celecoxib (5 mM) for in vitro use was made in absolute ethanol and dimethyl sulphoxide, respectively.

### CRC cell lines

CT26 and COX^low^-CT26 (CRISPR-Cas9 targeted deletion targeting *Ptgs1* and *Ptgs2*) mouse CRC cells were a gift from Dr Santiago Zelenay (Cancer Research UK Institute, Manchester, UK) [19]. MC38 mouse CRC cells were a gift from Professor Daniel Beauchamp (Vanderbilt University, TN) [20], from which we isolated a motile, EPA-resistant sub-population of MC38 cells, termed MC38r, using transforming growth factor (TGF)β (5 ng/ml) as a chemoattractant in a Transwell^®^ migration assay. MC38r cells have remained phenotypically stable during multiple passages over several years (Online Resource 1). All human CRC cells were obtained from the ATCC (https://www.atcc.org), except TC71 cells, which were a gift from Professor Alex Duval (INSERM, France). Human CRC cell lines were authenticated by STR profiling. All cell lines were tested regularly for *Mycoplasma* infection.

### Gene expression microarray analysis

Total RNA (100 ng) was processed according to the Illumina Whole-genome Gene Expression Direct Hybridization Assay Guide before 1.5 μg of cRNA was hybridised to a Mouse WG-6_v2 Beadchip as technical triplicates. Array 1 (Geo/GSE135607) compared gene expression in MC38 cells treated with 50 μM EPA-FFA for 24 h compared with MC38 cells exposed to the same dilution of ethanol (0.15% v/v) only. Array 2 (Geo/GSE135135) compared basal gene expression levels between MC38r and MC38 cells. Illumina GenomeStudio software v2010.2 and Ingenuity Pathway Analysis^®^ (December 2013 version) were used for data analysis.

### MTT assay

Cell sensitivity to EPA-FFA, aspirin and celecoxib was determined using the 3-(4,5-dimethylthiazol-2-yl)-2,5-diphenyltetrazolium bromide (MTT) cell viability assay [21]. Cells were incubated for 96 h with test agents, alone and in combination. The half-maximal inhibitory concentration (IC_50_) was calculated using either variable slope or biphasic equations in Prism6™ (GraphPad Software) and is expressed as the mean ± standard error of the mean (SEM).

### COX protein expression in mouse CRC cells

Total protein lysates (100 µg) were analysed by Western blotting using goat polyclonal antibodies against COX-1 (sc-1752, 1/250) and COX-2 (sc-1745, 1/250; both Santa Cruz, USA), as well as mouse monoclonal anti-β-actin antibody (clone AC-15, 1/10,000; Sigma-Aldrich, UK), incubated overnight at 4 °C. Secondary antibodies (1/2000, DakoCytomation, UK) were used for one hr at ambient temperature prior to visualisation using Supersignal West Pico Chemiluminescence (Pierce, UK).

### Real-time reverse transcription-polymerase chain reaction (RT-qPCR) measurement of *PTGS*1 and *PTGS*2 transcript levels

Total RNA was extracted using the RNeasy Micro kit (QIAGEN, UK). and then treated with DNase I (New England Biolabs, UK). cDNA synthesis was performed with 1 μg of total RNA input using the Omniscript RT kit (QIAGEN, UK). Real-time PCR was performed using GoTaq qPCR mastermix (Promega, UK) and the following primers, *PTGS1*: forward 5′-GAGCAGCTTTTCCAGACGA-3′ and reverse 5′-TCCTCGATGACAATCTTGATG-3′, *PTGS2*: forward 5′-CCCTTGGGTGTCAAAGGTAA-3′, reverse 5′-GCCCTCGCTTATGATCTGTC-3′ and *GAPDH*: forward 5′-TCAACGACCACTTTGTCAAGC-3′ and reverse 5′-CCAGGGGTCTTACTCCTTGG-3′. Relative quantification using ThermoFisher Connect™ generated a gene expression score for COX-1 and COX-2 according to the respective ∆Ct value [Ct_(PTGS)_ − Ct_(GAPDH)_] tertile [zero for no expression (as defined by the absence of a Ct_(PTGS)_ value below that associated with background), or 1–3]. The overall COX score for each cell line was determined by summing COX-1 and COX-2 scores. Data are expressed as the mean ± SEM of three biological replicates for each human CRC cell line.

### In vivo studies

Female CD1 Nude and *BALB/c* mice (Charles River Laboratories, UK) were housed in a specific pathogen-free environment. All experiments were undertaken with UK Home Office approval. Five-week-old mice were provided with isocaloric diets ad libitum for 14 days. Diets were based on a modified AIN-93G diet base, in which 7% (w/w) soybean oil was replaced by corn oil. EPA-TG {6.1% (w/w); equivalent to 5% (w/w) EPA-FFA content, for which we have previously demonstrated anti-CRC activity [8, 22]} replaced the equivalent amount of corn oil in EPA-containing diets. Aspirin-containing diets included either 200 parts per million (ppm), 400 ppm or 600 ppm aspirin. Experimental diets were manufactured by Safe (France), delivered in vacuum-packed 1 kg bags and stored at 4 °C to minimize oxidation. Food was replaced every 3–4 days, during which time there was no increase in lipid oxidation from baseline values as shown by dedicated stability testing (Online Resource 2). For subcutaneous tumour models, 10^7^ cells were injected subcutaneously into the flank of each CD1 Nude mouse on day 15. Animals continued on the same diet for a further 14 days until sacrifice. Tumour volume in mm^3^ (*π*/6 × [length × width]^3/2^) was measured using calipers. For the CRCLM model, 5 × 10^6^ CRC cells in 100 µl sterile PBS were introduced into the spleen by high-frequency ultrasound-guided injection (VEVO770, VisualSonics Inc., Canada) under isofluorane anaesthesia [8]. Mice continued on the same diet for a further 14 days until sacrifice. Tumour burden was determined by measuring liver weight at a sacrifice. Tissue samples were either fixed in 4% (w/v) paraformaldehyde in PBS overnight, prior to embedding in paraffin or were snap-frozen in liquid N_2_ before storage at − 80 °C. Urine and blood samples were also collected at a sacrifice. Serum was obtained after allowing the blood to clot at room temperature for 30 min, centrifugation at 2000*g* for 10 min at 4 °C, before storage at − 80 °C.

### Eicosanoid and fatty acid measurement

6-Keto-prostaglandin (PG) F_1α_, 11-dehydro-thromboxane (TX) B_2_, TXB_2_ and PGE_2_ levels were measured by specific immunoassays (Cayman Chemical, US).

Fatty acids were measured by liquid chromatography-electrospray ionization-tandem mass spectrometry (LC–ESI–MS/MS), as described previously [23]. Fatty acid levels are reported as the % of the total fatty acid content. For chiral analysis of 15-hydroxyeicosatetraenoic acid (HETE), HCA-7 human CRC cells were treated for 30 min with 500 µM aspirin (or equivalent DMSO control carrier) before the cell-conditioned medium was removed and refreshed with added 1 µM C20:4n6 arachidonic acid (AA) (or the equivalent v/v dilution of ethanol) for a further three hrs. Conditioned media were collected and analysed for 15-HETE by chiral LC–ESI–MS/MS, as described previously [24].

### Immunohistochemistry for COX-1 and COX-2

Sections (5 µm) were dewaxed and rehydrated before antigen retrieval (Access Revelation solution, Menarini Diagnostics, UK). Following a peroxidase block (Menarini Diagnostics), slides were blocked in casein solution (Menarini Diagnostics) and then incubated for 1 h at room temperature with either goat polyclonal anti-COX-1 (sc-1752, 1/100, Santa Cruz), or rabbit polyclonal anti-COX-2 (160162, 1/5, Cayman Chemical, UK) antibody, followed by addition of Impress™ Goat polymer or Impress™ Rabbit polymer (Vector Laboratories, UK) and DAB solution (Biocare Medical, UK), before counterstaining. Staining score of whole sections was determined by 2 independent observers blinded to treatment allocation on a scale of 0–2 for COX-1 and 0–3 for COX-2 (Online Resource 3, supplementary fig. S1).

## Results

### Differential sensitivity of human and mouse CRC cells to EPA

**Fig. 1:**
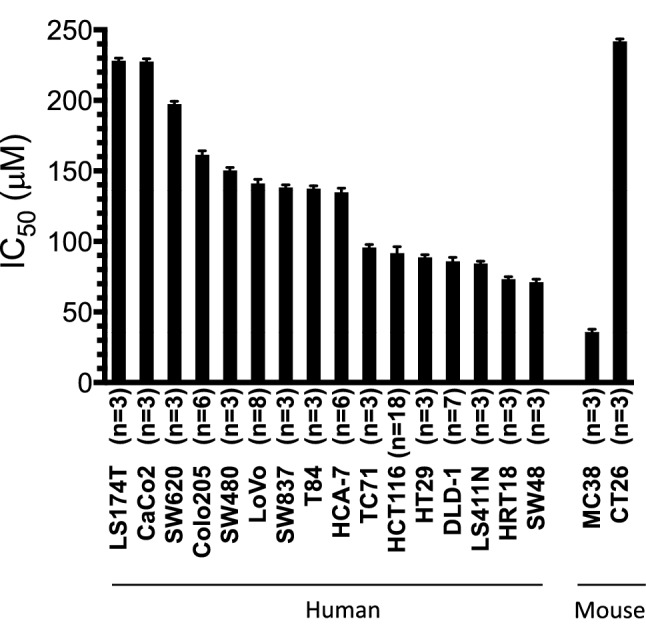
Colorectal cancer cell sensitivity to EPA in vitro. IC_50_ values for EPA after continuous exposure of human and mouse CRC cell lines to a range of EPA concentrations for 96 h. Data represent the mean ± SEM value for multiple independent replicates as indicated on the *x* axis. Human CRC cell lines are ordered according to decreasing mean IC_50_ value

We performed a comprehensive screen of EPA sensitivity of 16 human and 2 mouse CRC cell lines using an MTT cell viability assay. Human and mouse CRC cell lines displayed a range of sensitivity to EPA in vitro, with IC_50_ values ranging from 38.0 ± 3.4 µM (MC38 mouse CRC cells) to 241.8 ± 1.1 µM (CT26 mouse CRC cells; Fig. 1 and Online Resource 3—supplementary fig. S2). Several human CRC cell lines, including HRT18 and HT29 cells, exhibited a biphasic concentration–response relationship with EPA, which was evident as two distinct concentration–response phases separated by a plateau that was confirmed by regression analysis, suggesting that EPA may act via two separate mechanisms in some CRC cell lines (Online Resource 3—supplementary fig. S2). The EPA-sensitive mouse CRC cell line MC38 also displayed a biphasic response to EPA with component IC_50_ values of 10.5 ± 1.9 µM and 141.2 ± 1.3 µM (Online Resource 3—supplementary fig. S2).

### COX enzymes mediate EPA resistance in mouse CRC cells

**Fig. 2:**
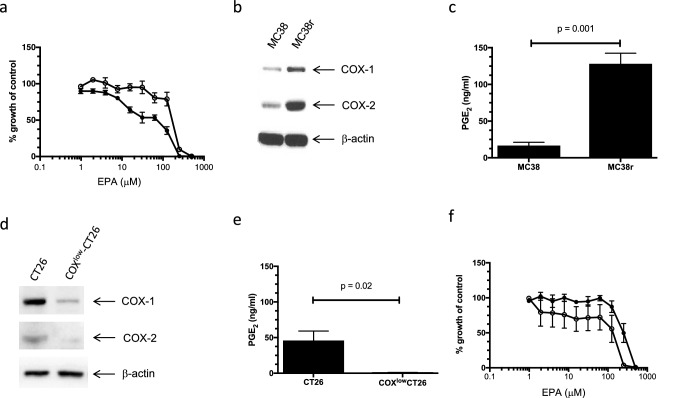
COX expression mediates EPA sensitivity of CRC cells in vitro. **a** MTT cell viability assay curves for the effect of EPA on MC38 (closed circles) and MC38r mouse CRC cells (open circles). Data represent the mean ± SEM % growth compared to untreated control cells for three independent replicates for each mouse CRC cell line. **b** Western blot analysis of COX isoform protein expression in MC38 and MC38r mouse CRC cells. **c** PGE_2_ levels in medium conditioned by MC38 and MC38r cells for 24 h. Data compared with Student’s *t* test. **d** Western blot analysis of COX isoform protein expression in CT26 and COX^low^-CT26 mouse CRC cells. **e** PGE_2_ levels in medium conditioned by CT26 and COX^low^-CT26 mouse CRC cells for 24 h. Data compared with Student’s *t* test. **f** MTT cell viability assay curves for the effect of EPA on CT26 (closed circles) and COX^low^-CT26 mouse CRC cells (open circles). Data represent the mean ± SEM % growth compared to untreated control cells for three independent replicates for each mouse CRC cell line. Two-way ANOVA confirmed a significant increase in EPA sensitivity of COX^low^-CT26 cells compared with CT26 mouse CRC cells (*p* = 0.03)

Relative EPA sensitivity of MC38 mouse CRC cells provided an opportunity to isolate EPA-resistant cells from the overall MC38 cell population. These cells, termed MC38r, were greater than 4-fold more resistant to EPA than parental MC38 cell cultures (Fig. 2a) and had lost the biphasic concentration–response displayed by MC38 cells (Fig. 2a). Relative EPA resistance of MC38r cells has been maintained for more than 30 passages (Online Resource 1), without selection pressure from exogenous EPA.

Differential gene expression analysis was performed using two whole-genome microarrays, which tested the effect of EPA exposure on MC38 cell gene expression (array 1) and compared basal gene expression in MC38r and MC38 cells (array 2). The full lists of differentially expressed genes are available in the Geo database. The highest differentially expressed genes included *Lox*, *Col6a1* and *P4ha1*, which are all linked to epithelial-mesenchymal transition and migration, likely resulting from the selection for the migratory phenotype of MC38r cells (Online Resource 4). The next three genes encoded an orphan G protein-coupled receptor (MRGPRF), a protein mediating inflammatory signalling (ANGPTL6), and COX-1 (Online Resource 4). There was no change in *Ptgs1* gene expression in upon MC38 exposure to EPA, however, its expression was increased 3.2-fold in the EPA-resistant MC38r cells compared to MC38 cells. Given existing data linking EPA activity and modulation of COX activity [25], we examined the expression of COX-1 and COX-2 proteins in MC38 and MC38r cells by western blotting. MC38r cells displayed an increase in COX-1 and COX-2 protein levels compared with MC38 cells (Fig. 2b), which was associated with a statistically significant increase in PGE_2_ production by MC38r cells compared with MC38 cells (Fig. 2c).

We next investigated whether there was a causal relationship between COX activity and CRC cell resistance to EPA. We examined a pair of isogenic mouse CRC cell lines; CT26, which is relatively resistant to EPA (Fig. 1), and COX^low^-CT26, in which expression and activity of both COX isoforms are reduced by CRISPR/Cas9 editing [19], thereby providing a genetic model of dual, non-selective COX inhibition that is achieved by NSAIDs such as aspirin and ibuprofen. Reduced COX-1 and COX-2 expression (Fig. 2d) and activity (Fig. 2e) in COX^low^-CT26 cells resulted in increased sensitivity to EPA (IC_50_ 148.9 ± 1.2 µM) compared with CT26 cells (IC_50_ 241.8 ± 1.1 µM; *p* = 0.03, 2-way ANOVA; Fig. 2f), thereby supporting the hypothesis that COX-1 and COX-2 mediate CRC cell resistance to EPA in vitro.

### Reduced COX activity is associated with enhanced EPA sensitivity of mouse CRC cell tumours in vivo

**Fig. 3:**
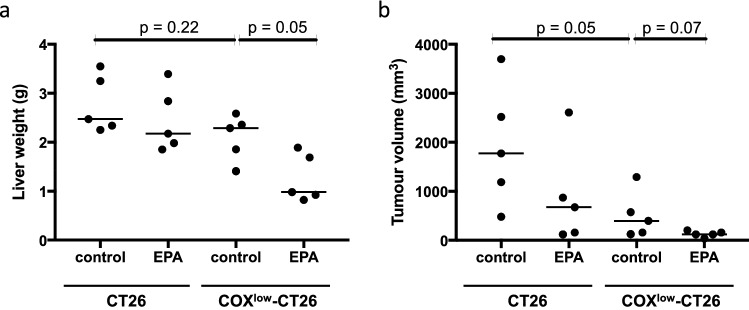
Reduced COX expression increases the sensitivity of mouse CT26 CRC cell tumours to EPA in vivo. **a** CT26 and COX^low^-CT26 cell liver metastases in *BALB/c* mice. The horizontal line indicates the median liver weight at a sacrifice in each group. The reference normal liver weight of female *BALB/c* mice is 0.99 g (https://www.jax.org/). **b** Subcutaneous CT26 and COX^low^-CT26 cell tumours in CD1 Nude mice receiving either control or EPA-containing diet (*n* = 5 per group). The horizontal line indicates the median tumour volume at a sacrifice in each group. Statistical significance was tested with the Mann–Whitney *U* test

We then tested whether reduced COX expression and activity in CRC cells would increase EPA sensitivity of CRC tumours in vivo. We used our established syngeneic *BALB/c*-CT26 model of CRCLM [8] to test the impact of oral administration of EPA on tumour growth. There was no significant reduction in COX^low^-CT26 cell tumour burden in mice receiving control diet compared with control animals with CT26 cell tumours, indicating that reduction of COX expression by CRC cells does not impact on tumour growth in an immunocompetent mouse tumour model (Fig. 3a). Oral EPA supplementation was associated with a 10% reduction in median liver weight in animals with CT26 cell liver metastases compared with a statistically significant 30% decrease in tumour burden (*p* = 0.05) associated with EPA treatment in mice bearing COX^low^-CT26 CRC cell liver metastases (Fig. 3a), despite similar tissue levels of EPA in both tumour types (Online Resource 3—supplementary fig. S3a). Therefore, we concluded that reduced COX-1 and COX-2 activity results in increased sensitivity of CT26 mouse CRC cells to EPA in vivo.

If COX activity promotes CRC cell resistance to EPA, one might expect a relationship between COX-1/COX-2 expression and EPA sensitivity in individual CRC cell lines. No simple relationship between COX mRNA levels and the IC_50_ value for EPA measured by the MTT assay was evident in our large panel of human CRC cell lines (Online Resource 3—supplementary fig. S4a). Nevertheless, we observed that human CRC cell lines that demonstrated a biphasic concentration–response relationship with EPA had, in general, a lower COX-2 (not COX-1) score than counterparts with a mono-phasic relationship between growth and EPA concentration (Online Resource 3- supplementary fig. S4b). Furthermore, fatty acid analysis of human CRC cell lines treated with 5 µM EPA-FFA demonstrated a correlation between the fold-increase in % EPA content from baseline values and sensitivity to EPA (measured as a lower IC_50_ value) in the individual human CRC cell lines (Online Resource 3—supplementary fig. S5).

### COX enzymes modulate the direct anti-neoplastic activity of EPA on CRC cells

Zelenay and colleagues have reported that COX-2-PGE_2_ signalling represses the host anti-tumour response to subcutaneous CT26 mouse CRC cell tumours and that COX inhibitors have anti-tumour effects in this model through de-repression of the adaptive anti-tumour immune response [19]. Therefore, to determine the relative contributions of (1) modulation of the host anti-tumour immune response and/or (2) direct effects on tumour cell resistance by the COX enzymes that might contribute to EPA sensitivity of CRC cell tumours in vivo, we used CD1 Nude mice, which do not mount a T cell-dependent adaptive immune response. Consistent with the *BALB/c* mouse experiments, CT26 and COX^low^-CT26 tumour tissues incorporated EPA to a similar degree following dietary EPA administration (Online Resource 3- supplementary fig. S3b). COX^low^-CT26 cell tumours were significantly smaller than CT26 cell tumours suggesting that COX activity drives CT26 cell tumour growth directly in this subcutaneous tumour microenvironment (Fig. 3b). Dietary EPA treatment of CT26 cell tumour-bearing mice resulted in a 62% reduction in tumour growth [median tumour volume 676 mm^3^ (range 122–2609 mm^3^)] compared to diet controls [1776 mm^3^ (range 482–3699 mm^3^)] (Fig. 3b). However, EPA supplementation in COX^low^-CT26 cell tumour-bearing animals resulted in a more obvious decrease in tumour size, with uniform growth suppression of all subcutaneous tumours [median 122 mm^3^ (range 39–204 mm^3^)], compared with animals that received control diet [398 mm^3^ (range 125–1291 mm^3^), *p* = 0.07; Fig. 3b]. Consistent with our in vitro data, the efficacy of EPA in the immunodeficient CD1 Nude mouse model implies that the COX isoforms modify the response of tumour cells themselves to EPA in vivo, rather than modification of the host anti-tumour T cell-mediated immune response.

### Aspirin and celecoxib increase CRC cell sensitivity to EPA in vitro

Aspirin has known anti-CRC activity and is currently undergoing phase 3 RCT evaluation as an adjunct to standard care in individuals with CRC after surgical resection of the primary tumour (ClinicalTrials.gov NCT02804815) [26, 27]. Aspirin irreversibly inhibits COX-1 activity and modifies COX-2 activity leading to reduced PGE_2_ synthesis [28], thus providing dual COX inhibition.

**Fig. 4:**
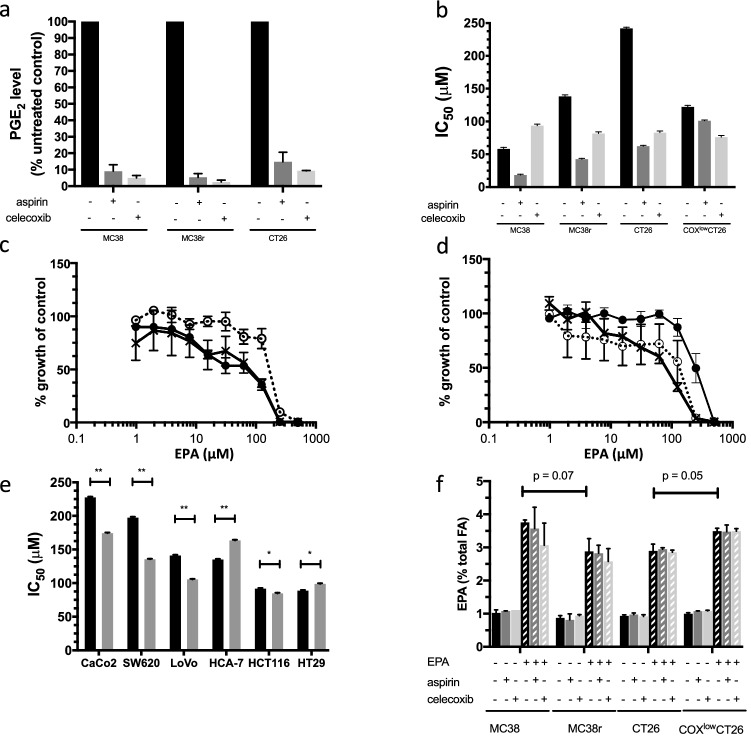
Aspirin increases EPA sensitivity of CRC cells in vitro. **a** PGE_2_ synthesis by mouse CRC cells in the presence or absence of aspirin (500 µM) or celecoxib (0.5 µM). Data represent the mean ± SEM % PGE_2_ level of three independent replicates compared with control cells. **b** Mouse CRC cell sensitivity to EPA in the presence or absence of aspirin (500 µM) or celecoxib (0.5 µM). Data represent the mean ± SEM IC_50_ values for three independent replicates. The differences in IC_50_ values for the combination treatments compared with EPA alone were statistically significant (Student’s *t* test *p* < 0.01). **c** Concentration–response relationship with EPA for MC38 (closed circles), MC38r (open circles) and aspirin-treated MC38r cells (crosses). Data are expressed as the mean ± SEM % growth compared with untreated control cells for three independent experiments. **d** Concentration–response relationship with EPA for CT26 cells (closed circles), COX^low^-CT26 cells (open circles) and aspirin-treated CT26 cells (crosses). Data are expressed as the mean ± SEM % growth compared to untreated control cells for 3 independent experiments. **e** Response to EPA of human CRC cell lines in the presence or absence of aspirin (500 µM). Data represent the mean ± SEM IC_50_ value for 3 independent replicates. The differences in IC_50_ values for the combination treatments compared with EPA alone were all statistically significant (Student’s *t* test *p* < 0.05). **f** EPA content (expressed as the % total fatty acids) of mouse CRC cells with or without the addition of 5 µM EPA to the culture medium, in the presence or absence of either aspirin (500 µM) or celecoxib (0.5 µM). Data represent the mean + SEM % EPA level of 3 independent replicates. In the absence of an error bar, data represent *n* = 1 as other samples failed analysis for technical reasons

To maximise the effect of aspirin on COX metabolism whilst avoiding any non-specific cytotoxic activity against CRC cells in vitro, we selected a dose of 500 μM aspirin to be used in combination with a range of EPA concentrations (Online Resource 3—Supplementary fig. S6a). Many other studies, which have previously investigated the effect of aspirin on CRC cell growth in vitro, have used higher concentrations of aspirin, often reaching 1–10 mM [29, 30]. Reduced PGE_2_ production in high COX-2-expressing MC38r and CT26 cells following 500 µM aspirin exposure confirmed effective COX-1 and COX-2 inhibition (Fig. 4a). In addition, we confirmed that 500 µM aspirin specifically altered COX-2 activity by demonstrating a switch in the chirality of the minor COX-2 product 15- HETE from the *S*- to the *R*-enantiomer that is known to occur after serine acetylation of the COX-2 active site, thus confirming the pharmacological specificity of our in vitro cell model of aspirin-induced COX inhibition (Online Resource 3—supplementary fig. S6b) [31].

Aspirin (500 µM) increased mouse CRC cell sensitivity to EPA, as measured by the MTT assay, in each cell line except for COX^low^-CT26 cells (Fig. 4b). In the presence of aspirin, the IC_50_ in MC38 cells was decreased nearly two-fold, whilst the IC_50_ for MC38r and CT26 cells, which express high levels of COX-2, was nearly four-fold lower in the presence of aspirin than for EPA alone (Fig. 4b). Aspirin restored the biphasic concentration–response relationship for EPA, which we had observed in MC38 cells (Fig. 2a), to MC38r cells (Fig. 4c). In the presence of aspirin, CT26 cells also displayed a biphasic concentration–response relationship with EPA, mimicking the difference between CT26 cells and COX^low^-CT26 cells (Fig. 4d). Three (CaCo2, SW620 and LoVo) of the six human CRC cell lines that we examined were more sensitive to EPA in the presence of aspirin by approximately 25% (Fig. 4e). The three human CRC cell lines that displayed increased EPA sensitivity in the presence of aspirin were all cell lines exhibiting relative EPA resistance (IC_50_ > 140 µM; Fig. 1).

We also demonstrated that the selective COX-2 inhibitor celecoxib, at a concentration (0.5 µM) that inhibited PGE_2_ production by > 90% in mouse CRC cell lines (Fig. 4a) and did not display toxicity (Online Resource 3—supplementary fig. S7), increased sensitivity of COX-2-expressing mouse CRC cells to EPA (Fig. 4b).

One hypothesis is that genetic or pharmacological inhibition of COX activity increases the sensitivity of CRC cells to EPA via a reduction in EPA catabolism and a consequent increase in cellular EPA level. Therefore, we investigated the effect of aspirin and celecoxib on cellular EPA levels in the absence and presence of exogenous EPA. As expected, the addition of EPA was associated with an increase in the proportion of EPA present in the total fatty acid pool in mouse CRC cells (Fig. 4f). It is noteworthy that increased COX-2 expression in MC38r cells was associated with reduced EPA levels compared with MC38 cells (Fig. 4f) and that COX^low^-CT26 cells displayed increased EPA levels compared with CT26 cells, which express higher levels of COX-1 and COX-2 (Fig. 4f). However, pharmacological COX inhibition by either aspirin or celecoxib was not associated with an increase in cellular EPA content, even in the presence of exogenous EPA (Fig. 4f).

Supplementary fig. S8 (Online Resource 3) demonstrates that the mouse CRC cell lines were each capable of conversion of EPA to *n*-3 docosapentaenoic acid (DPA), but that this was not associated with desaturation to DHA or displacement of n-6 arachidonic acid (AA) from the cellular fatty acid pool.

We conclude that alteration of cellular EPA levels and/or the metabolic fate of EPA is not likely to explain simply how a reduction in COX activity by genetic or pharmacological means is associated with increased sensitivity of CRC cells to EPA.

### Anti-CRC activity of EPA in vivo is not enhanced by aspirin dosing that mimics low-dose aspirin use in humans

Prior to testing the effect of aspirin on EPA sensitivity of CRC cell tumours in vivo, we confirmed that our mouse model reflected the pharmacodynamic profile of low-dose (≤ 300 mg daily) aspirin use in humans. A dose-dependent reduction in serum TXB_2_ level (a measure of platelet COX-1 inhibition) was evident (Online Resource 3—supplementary fig. S9a). Administration of a 600 ppm aspirin-containing diet for 9 days was associated with a reduction in serum TXB_2_ levels of greater than 85% in non-tumour bearing CD1 Nude mice (median 13.3 ng/ml (range 3.5–51.7 ng/ml) compared with animals that were fed a control diet [122 ng/ml (range 31.4–131.5); Online Resource 3—supplementary fig. S9a]. This mirrors the percentage reduction in serum TXB_2_ observed in humans following dosing with aspirin 75 mg daily [32]. The diet containing 600 ppm aspirin was well tolerated based on body weight monitoring (Online Resource 3—supplementary fig. S9b).

**Fig. 5:**
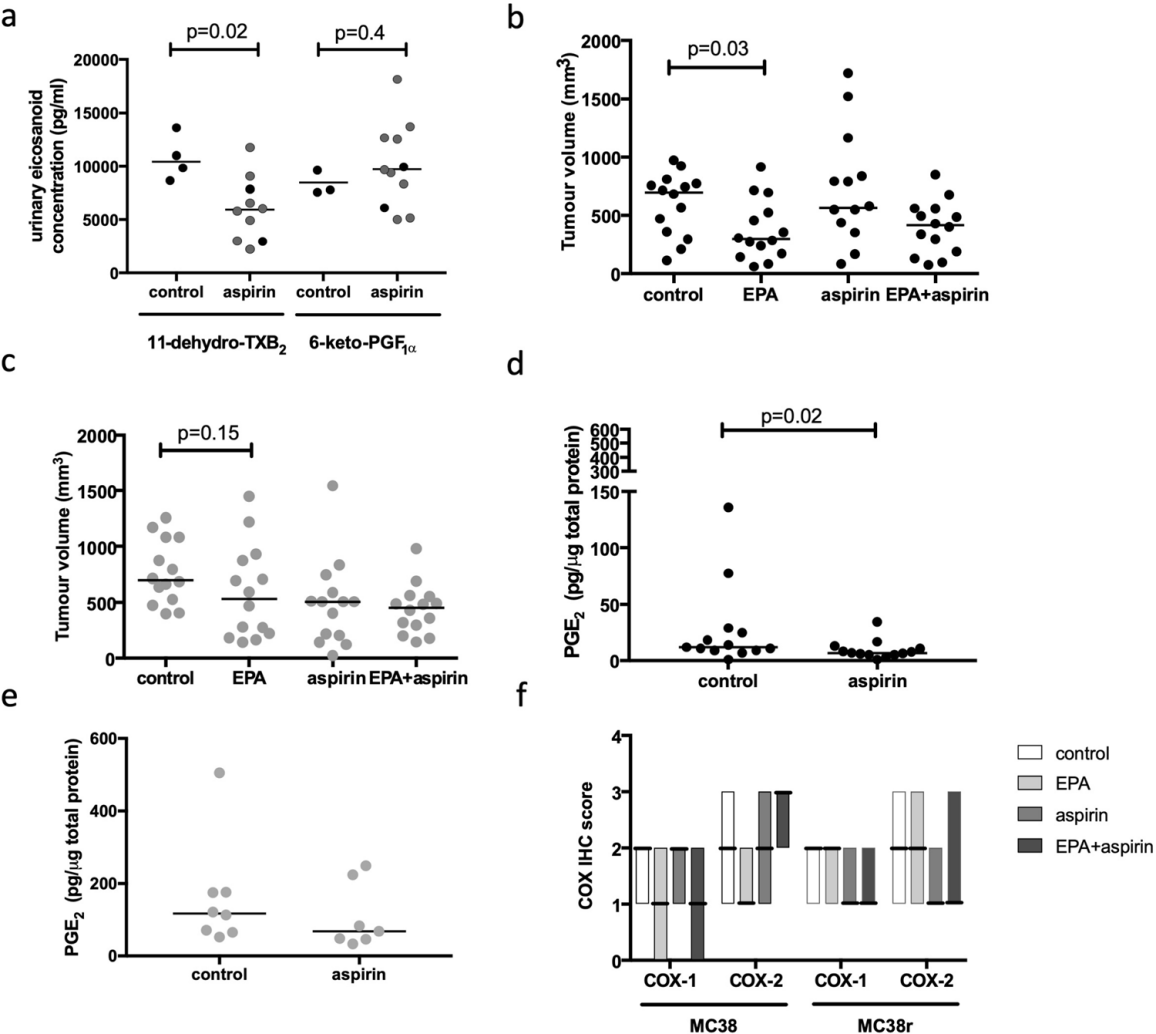
Aspirin treatment does not improve MC38 CRC cell tumour response to EPA in a mouse model that mimics low-dose aspirin use in humans. **a** Effect of oral aspirin dosing on levels of the urinary metabolites 11-dehydro-TXB_2_ and 6-keto-PGF_1α_ in CD1 Nude mice with MC38 cell (black circles) or MC38r cell (grey circles) tumours. Urine was not obtained from all animals. The horizontal line indicates the median value for each group. Statistical significance was tested using the Mann–Whitney *U* test. **b** MC38 cell and **c** MC38r cell tumour volume in CD1 Nude mice receiving diets containing either EPA-TG (6.1%), aspirin (600 ppm), or a combination of EPA and aspirin, or control diet (*n* = 14 per group). The line indicates the median value for each group. Statistical significance was tested using the Mann–Whitney *U* test. **d** MC38 cell and **e** MC38r cell tumour PGE_2_ levels in animals fed either control or aspirin-containing diet. The line indicates the median value for each group. **f** COX-1 and COX-2 protein expression measured by immunohistochemistry in MC38 and MC38r tumours. The bars delineate the range of IHC scores obtained within a group and the bold line indicates the median score for each group

MC38 and MC38r mouse CRC cells were grown as subcutaneous tumours in CD1 Nude mice that received either control diet, a diet supplemented with 6.1% (w/w) EPA-TG alone, 600 ppm aspirin-containing diet, or a diet containing both EPA and aspirin (*n* = 10 each group). We confirmed that there was a significant reduction in urinary 11-dehydro-TXB_2_ level (indicative of effective systemic COX-1 inhibition) at sacrifice in animals treated with aspirin alone compared with controls (Fig. 5a). However, there was no significant reduction in urinary 6-keto-PGF_1α_ (the stable hydrolysed product of COX-2-derived endothelial cell PGI_2_ production) levels associated with administration of 600 ppm aspirin-containing diet for 28 days (Fig. 5a), suggesting ineffective systemic COX-2 inhibition in CD1 Nude mice, which is consistent with the pharmacodynamic profile of low-dose aspirin use in humans [33].

Dietary EPA supplementation alone resulted in a significant reduction in MC38 cell tumour volume [median 297 mm^3^ (range 62–918 mm^3^)] compared with control tumours [699 mm^3^ (range 116–974 mm^3^); *p* = 0.03; Fig. 5b]. Addition of aspirin to the EPA-containing diet did not further reduce MC38 cell tumour size compared with EPA alone (Fig. 5b). Treatment with EPA alone did not reduce MC38r cell tumour volume (*p* = 0.15; Fig. 5c), but combined treatment with EPA and aspirin did result in a significant reduction in MC38r tumour volume [median 455 mm^3^ (range 146–980 mm^3^)] compared with tumours from mice that received control diet [median 699 mm^3^ (range 400–1258) mm^3^; Fig. 5c]. However, this was indistinguishable from the MC38 tumour sizes observed in mice treated with aspirin alone (Fig. 5c). Levels of EPA measured in control and EPA-treated MC38 and MC38r cell tumours were comparable (Online Resource 3—supplementary fig. S10). The combination of aspirin and EPA was associated with a statistically significant increase (*p* = 0.01) in tumour EPA content compared with EPA alone in MC38 cell tumours, but not MC38r cell tumours (Online Resource 3—supplementary fig. S10). In keeping with dominant inhibition of COX-1 activity by aspirin, compared with COX-2, in the CD1 Nude mouse model, intra-tumoral PGE_2_ levels were reduced only partially by aspirin in both MC38 and MC38r cell tumours (Fig. 5d, e), with an attenuated, statistically insignificant response in MC38r cell tumours, in which COX-2-dependent PGE_2_ synthesis is increased (Fig. 5d, e). Reduction in tumour PGE_2_ levels was not explained by decreased COX-1 and COX-2 protein levels, as there was no significant difference in expression of either COX-1 or COX-2 protein in either MC38 or MC38r cell tumours in any of the treatment groups (Fig. 5f). We conclude that treatment with aspirin, in a mouse model mimicking low-dose aspirin use in humans, does not increase the sensitivity of CRC cell tumours to EPA, in direct contrast to the effects seen in vitro.

**Fig. 6:**
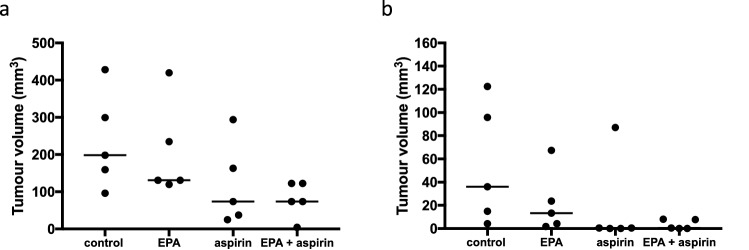
Effect of EPA and aspirin on human CRC cell tumour growth in vivo. **a** SW620 and **b** HCA-7 human CRC cells were grown as subcutaneous tumours in CD1 Nude mice provided with a control diet or a diet containing either EPA (6.1%), aspirin (600 ppm) or a combination of EPA and aspirin (*n* = 5 per group). The line indicates the median tumour volume for each group

We also tested the effect of aspirin co-treatment on human CRC cell sensitivity to EPA in vivo. We examined SW620 and HCA-7 human CRC cell xenograft tumours in CD1 Nude mice as exemplar EPA-resistant and -sensitive human CRC cell lines, which both displayed similar sensitivity to EPA as their in vitro IC_50_ values would suggest (Figs. 1, 4e), with faster-growing SW620 cell tumours being resistant to EPA-TG treatment alone (Fig. 6a) compared with HCA-7 cell tumours (Fig. 6b), despite similar EPA incorporation in each tumour-type when animals were provided with an EPA-containing diet (Online Resource 3—supplementary fig. S11). In both CRC cell models, aspirin treatment alone reduced tumour growth, with no additional benefit of combined EPA and aspirin treatment observed (Fig. 6).

## Discussion

We demonstrate that COX-1 and COX-2 drive EPA resistance of CRC cells and that the widely used, non-selective COX inhibitor aspirin and selective COX-2 inhibitor celecoxib mirror the effect of genetic ablation of COX-1 and COX-2 by sensitising multiple human and mouse CRC cell lines to EPA in vitro. Previous reports have demonstrated that the combination of an O3FA with a COX inhibitor in vitro provides synergistic anti-neoplastic benefit. For example, a combination of the selective COX-2 inhibitor celecoxib and DHA led to decreased proliferation and increased apoptosis of HCA-7 human CRC cells [34]. Moreover, combined DHA and sulindac (a non-selective COX inhibitor) treatment decreased growth and increased apoptosis of three human CRC cell lines compared with either agent alone [35].

Aspirin dosing in our CD1 Nude mouse model [36, 37], which mimicked low-dose aspirin use in humans, based on profound platelet COX-1 inhibition but an absence of systemic COX-2 inhibition, did not increase the sensitivity of MC38 mouse CRC cell tumours to EPA in vivo, in direct contrast to the corresponding in vitro data. We demonstrated that there was inefficient inhibition of PGE_2_ production in CRC cell tumour tissue, despite efficient systemic COX-1 inhibition. Therefore, a plausible explanation for the lack of aspirin efficacy in vivo is that there was incomplete intra-tumoral COX inhibition by aspirin, in contrast to the effective dual COX inhibition obtained by either genetic deletion or pharmacological inhibition in vitro.

Importantly, the mode of administration of aspirin in rodent models may affect systemic COX inhibition. Tissue PGE_2_ levels in studies comparing oral gavage and dietary administration of aspirin and the NSAID sulindac are lower after oral gavage, despite equivalent platelet COX-1 inhibition, suggesting that the duration and extent of tumour COX-2 inhibition in mice are highly dependent on the dosing route and schedule [38, 39].

A preliminary observation from the seAFOod polyp prevention trial is that combined treatment with aspirin (300 mg once daily) and EPA (2 g FFA daily) reduced colorectal adenoma recurrence (as measured by the number of colorectal adenomas per participant) to a greater extent than either EPA or aspirin alone [6], which is consistent with our in vitro observations. Ongoing work is exploring whether aspirin use in the seAFOod Trial was associated with significant inhibition of systemic and target tissue (rectal mucosa) PGE_2_ synthesis by measuring urinary PGE-M and rectal mucosal PGE_2_ levels, respectively.

It is recognised that 18*R*-hydroxyeicosapentaenoic acid production by aspirin-acetylated COX-2 can lead to 5-lipoxygenase-dependent synthesis of E-type resolvins (Rv), which have anti-neoplastic activity [40]. The hypothesis that RvE1 and/or RvE2 increase sensitivity of CRC cells to EPA is valid and testable in future experiments.

We also conclude that COX-1 and COX-2 modulate resistance to EPA by altering sensitivity of CRC cells themselves to EPA, rather than by a mechanism including enhancement of the host anti-tumour immune response. Our data demonstrating that loss of CT26 CRC cell COX expression has similar effects on the anti-tumour activity of EPA in both immunocompetent *BALB/c* and immunocompromised CD1 Nude mice contrast with the study of Zelenay et al., in which genetic and pharmacological COX inhibition (in this case using the selective COX-2 inhibitor celecoxib) reduced CT26 cell tumour growth in immunocompetent, but not immunocompromised mice [19]. The difference might be due to differences in Nude mice from different colonies. Consistent with our data, several other studies have indicated that COX inhibition reduces growth of multiple tumour types in immunodeficient nude mice [40–43].

The mechanism whereby COX expression drives CRC cell resistance to EPA remains unclear. One hypothesis is that EPA acts via a COX-independent mechanism(s), which is dependent on the intracellular EPA level gained, that is, in turn, controlled by the degree of EPA metabolism by COX enzymes. The relationship between cellular EPA levels at baseline and in response to exogenous EPA in CRC cell lines with different levels of COX expression is consistent with this hypothesis. However, pharmacological COX inhibition was not associated with increased cellular EPA content and there was no discernible relationship between COX expression and EPA sensitivity (as measured by the MTT assay) across the panel of human CRC cell lines. It is possible that a large number of catabolic fates of EPA (via multiple oxygenases other than COX, as well as mitochondrial beta-oxidation) obscures the contribution of the COX enzymes to cellular EPA content and response to supplementation unless there are large changes in COX expression and activity as observed in a CRISPR/Cas9 recombination model. The intriguing finding that some CRC cells display a biphasic concentration–response relationship with EPA suggests that EPA has at least two separate mechanisms of anti-CRC activity. The observation that genetic loss of, or pharmacological inhibition of, COX leads to the emergence of a biphasic concentration–response relationship in EPA-resistant CRC cells suggests that COX activity may ordinarily repress an EPA target in CRC cells that is then unmasked, leading to increased CRC cell sensitivity to EPA.

In conclusion, COX activity drives EPA resistance in CRC cells providing a clinically relevant opportunity to enhance the anti-neoplastic activity of EPA by pharmacological inhibition of COX using agents already in clinical use. The inability of aspirin to enhance the anti-CRC activity of EPA in vivo, in direct contrast to the in vitro data and preliminary seAFOod polyp prevention trial observations, suggests that the degree of tumour tissue COX inhibition achieved by aspirin is likely to be critical for its ability to augment the anti-CRC activity of EPA.

## Electronic supplementary material

Below is the link to the electronic supplementary material.

Supplementary file1 (PDF 149 kb)

Supplementary file2 (PDF 101 kb)

Supplementary file3 (PDF 4377 kb)

Supplementary file4 (PDF 90 kb)

## Data Availability

Illumina GenomeStudio software v2010.2 for intensity array analysis and Ingenuity Pathway Analysis^®^ (December 2013 version) was used for pathway and function analysis of gene lists.

## Data availability

Data are publicly available in the Geo database (https://www.ncbi.nlm.nih.gov/geo/) under reference GSE135607 (array 1) and GSE135135 (array 2).
