# Supplementary material for: Cyclooxygenase activity mediates colorectal cancer cell resistance to the omega-3 polyunsaturated fatty acid eicosapentaenoic acid
Source: Cancer Chemother Pharmacol. 2020 Oct 11;87(2):173–84. doi: 10.1007/s00280-020-04157-2 (PMC7870614; doi:10.1007/s00280-020-04157-2)
Supplement: Supplementary file 1 — Supplementary file1 (PDF 149 kb) [file 280_2020_4157_MOESM1_ESM.pdf]

Cyclooxygenase activity mediates colorectal cancer cell resistance to the omega-3 polyunsaturated fatty acid eicosapentaenoic acid. Cancer Chemotherapy and Pharmacology.

Milene Volpato, Nicola Ingram, Sarah L Perry, Jade Spencer, Amanda D Race, Catriona Marshall, John M Hutchinson, Anna Nicolaou, Paul M Loadman, P Louise Coletta and Mark A Hull.

Corresponding author contact: Leeds Institute of Medical Research at St James's, University of Leeds, St James's University Hospital, Leeds LS9 7TF, United Kingdom, m.volpato@leeds.ac.uk

Table S1: MC38r resistance to EPA is stable over time. IC_{50} values were obtained for MC38r cells over time (38 passages), using the MTT assay following 96 hr exposure to EPA-FFA.

Time post MC38r cell isolation (months)	IC_{50} (mean ± SEM)*
1	163.2 ± 1.0 (n=3)
9	146.7 ± 1.3 (n=3)
15	135.3 ± 1.2 (n=6)
21	145.3 ± 1.4 (n=3)
33	138.5 ± 1.2 (n=3)
41	172.4 ± 1.1 (n=2)
51	184.1 ± 1.1 (n=7)

*IC_{50} values are µM
