# Supplementary material for: Cyclooxygenase activity mediates colorectal cancer cell resistance to the omega-3 polyunsaturated fatty acid eicosapentaenoic acid
Source: Cancer Chemother Pharmacol. 2020 Oct 11;87(2):173–84. doi: 10.1007/s00280-020-04157-2 (PMC7870614; doi:10.1007/s00280-020-04157-2)
Supplement: Supplementary file 2 — Supplementary file2 (PDF 101 kb) [file 280_2020_4157_MOESM2_ESM.pdf]

Cyclooxygenase activity mediates colorectal cancer cell resistance to the omega-3 polyunsaturated fatty acid eicosapentaenoic acid. Cancer Chemotherapy and Pharmacology.

Milene Volpato, Nicola Ingram, Sarah L Perry, Jade Spencer, Amanda D Race, Catriona Marshall, John M Hutchinson, Anna Nicolaou, Paul M Loadman, P Louise Coletta and Mark A Hull.

Corresponding author contact: Leeds Institute of Medical Research at St James's, University of Leeds, St James's University Hospital, Leeds LS9 7TF, United Kingdom, m.volpato@leeds.ac.uk

Table S2: Stability of EPA-TG in AIN-93G diet. Vacuumed-packed bags were opened and stored at either 4°C or room temperature. Samples were analysed by CRODA (UK).
The peroxide value (PoV) represents lipid oxidation and is expressed as millimoles of active oxygen per kg lipid. The p-Anisidine Value (pAV) corresponds to the quantity of aldehydes and ketones generated from secondary oxidation of polyunsaturated fatty acids. pAV is determined as the absorbance at 366 nm in AV units.

	Storage at 4°C		Storage at room temperature	
Time from opening diet bag (days)	PoV	pAV	PoV	pAV
Baseline	21.7	5.0	21.7	5.0
1	19.5	5.2	17.9	4.0
3	19.3	4.4	17.9	4.1
7	18.6	4.3	23.8	4.7
14	19.3	4.9	52.3	8.5
28	21.0	4.7	>90.4*	19.2
56	22.4	4.5	-	-
84	25.5	5.2	-	-

*glassware would not accommodate more titrant
