# Supplementary material for: Cyclooxygenase activity mediates colorectal cancer cell resistance to the omega-3 polyunsaturated fatty acid eicosapentaenoic acid
Source: Cancer Chemother Pharmacol. 2020 Oct 11;87(2):173–84. doi: 10.1007/s00280-020-04157-2 (PMC7870614; doi:10.1007/s00280-020-04157-2)
Supplement: Supplementary file 3 — Supplementary file3 (PDF 4377 kb) [file 280_2020_4157_MOESM3_ESM.pdf]

Cyclooxygenase activity mediates colorectal cancer cell resistance to the omega-3 polyunsaturated fatty acid eicosapentaenoic acid.

Cancer Chemotherapy and Pharmacology

Milene Volpato, Nicola Ingram, Sarah L Perry, Jade Spencer, Amanda D Race, Catriona Marshall, John M Hutchinson, Anna Nicolaou, Paul M Loadman, P Louise Coletta and Mark A Hull

Corresponding author contact:

Leeds Institute of Medical Research at St James's, University of Leeds, St James's University Hospital, Leeds LS9 7TF, United Kingdom, m.volpato@leeds.ac.uk

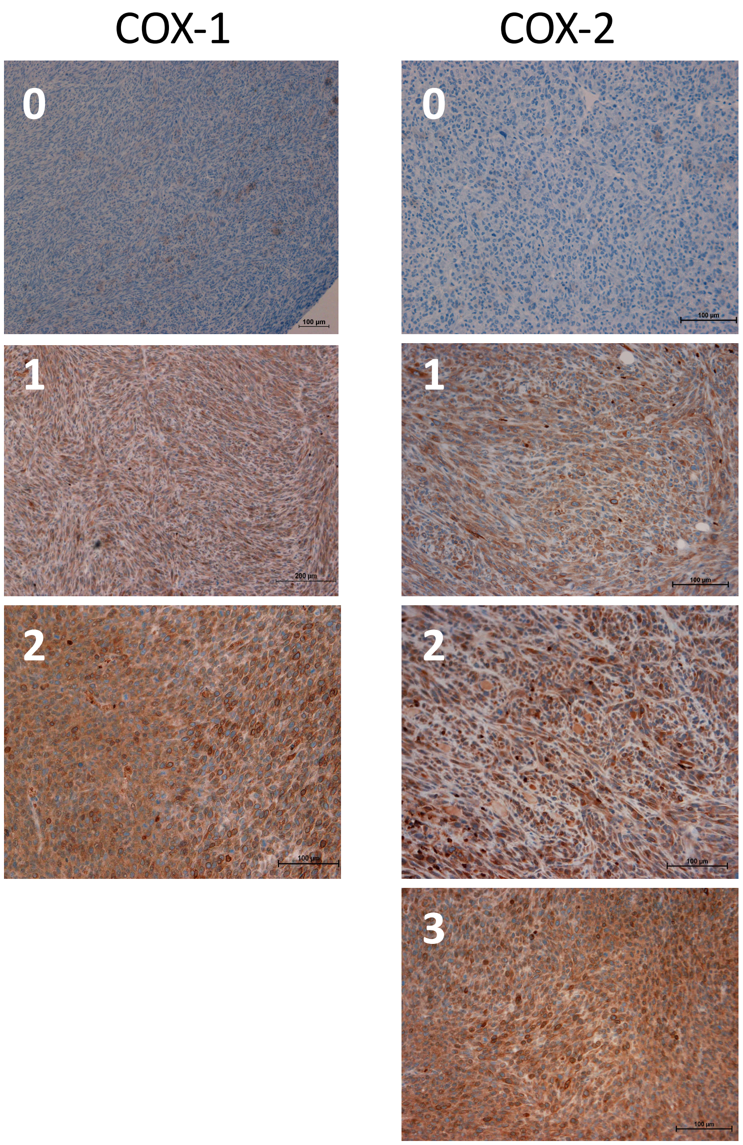

Supplementary fig. S1: Representative photomicrographs from COX immunohistochemistry on subcutaneous mouse CRC cell tumours in CD1-nude mice. COX-1 and COX-2 scores corresponding to staining intensity are noted in each panel.

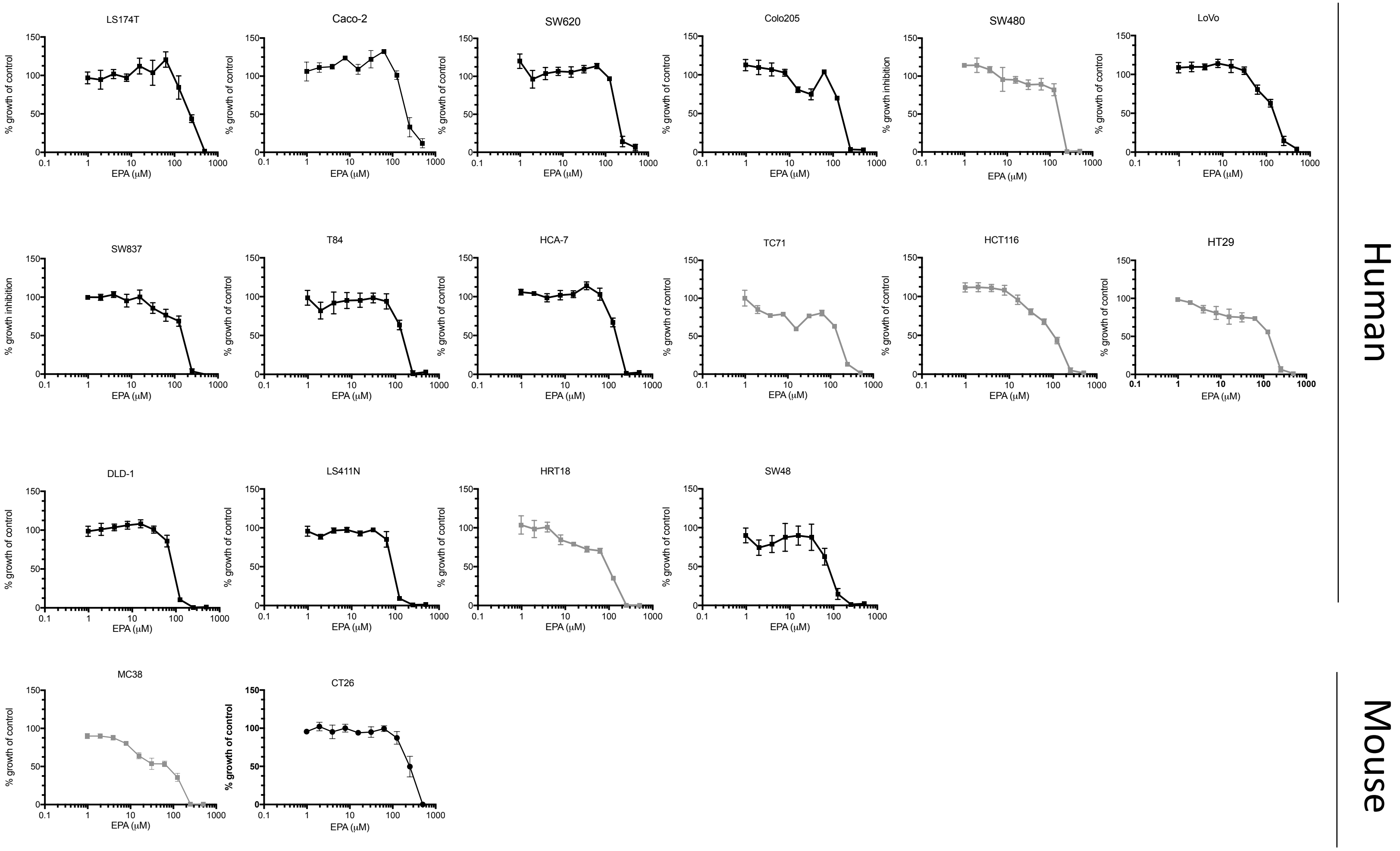

Supplementary fig. S2: Individual CRC cell concentration-response curves for EPA measured by a MTT cell viability assay in order of decreased IC_{50} values. Data in grey highlight biphasic dose-response curves defined by Prism regression analysis. Data represent the mean ± SEM % growth relative to untreated control cells for a minimum of 3 independent replicates (see figure 1).

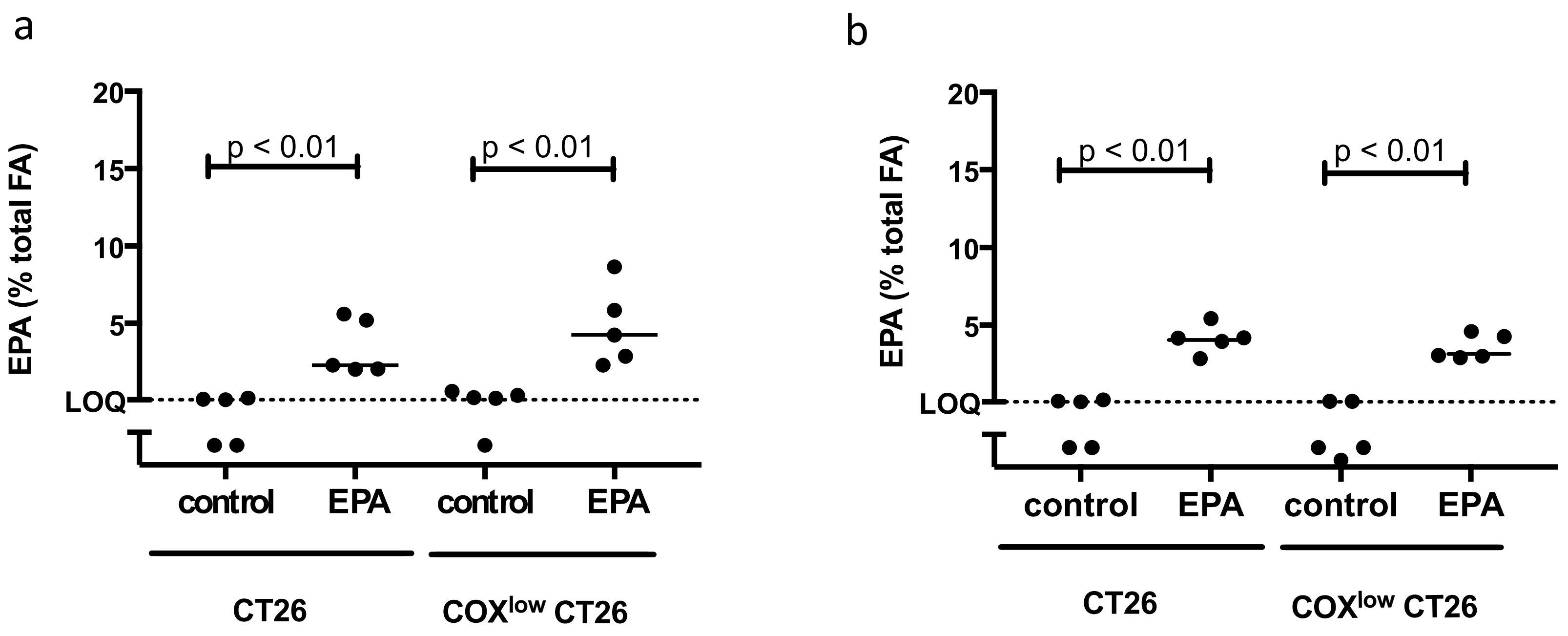

Supplementary fig. S3: EPA levels in mouse CT26 CRC cell tumour tissue. a) CT26 and COX^{low}-CT26 mouse CRC cells were grown as liver metastases in *BALB/c* mice receiving a control or EPA-supplemented diet (n=5 per group). b) CT26 and COX^{low}-CT26 cells were grown as subcutaneous tumours in CD1-nude mice fed a control or EPA-supplemented diet (n=5 per group). The line indicates the median EPA value for each group. The limit of quantification (LOQ) for EPA was 0.06% of total tissue fatty acids. P values are for the Mann-Whitney U test.

a

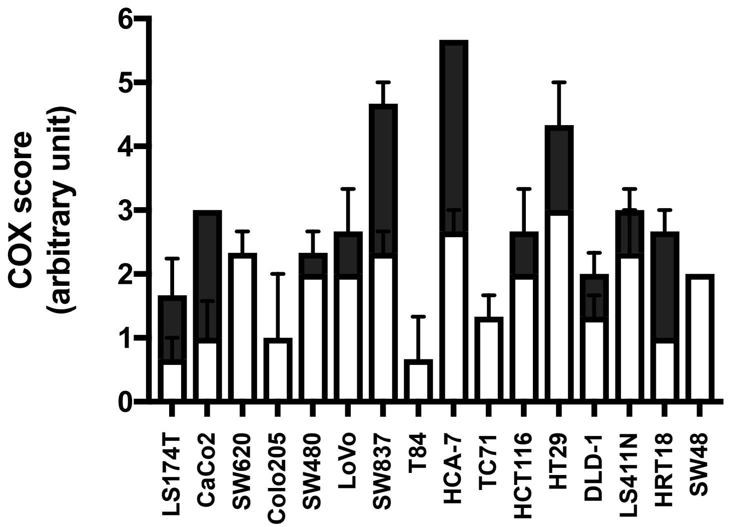

b

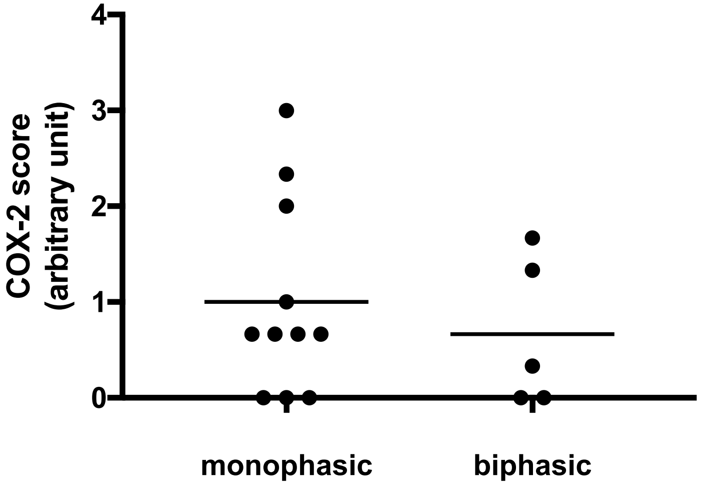

Supplementary fig. S4: COX-1 and COX-2 expression in human CRC cell lines. a) The COX score (a composite of COX-1 and COX-2 scores was derived from ΔC_t tertiles for each gene using *GAPDH* as the reference gene (see *Methods*). The cell lines are in order of decreasing IC_{50} for EPA (fig.1). b) Comparison of COX-2 expression levels in monophasic and biphasic dose-response curves.

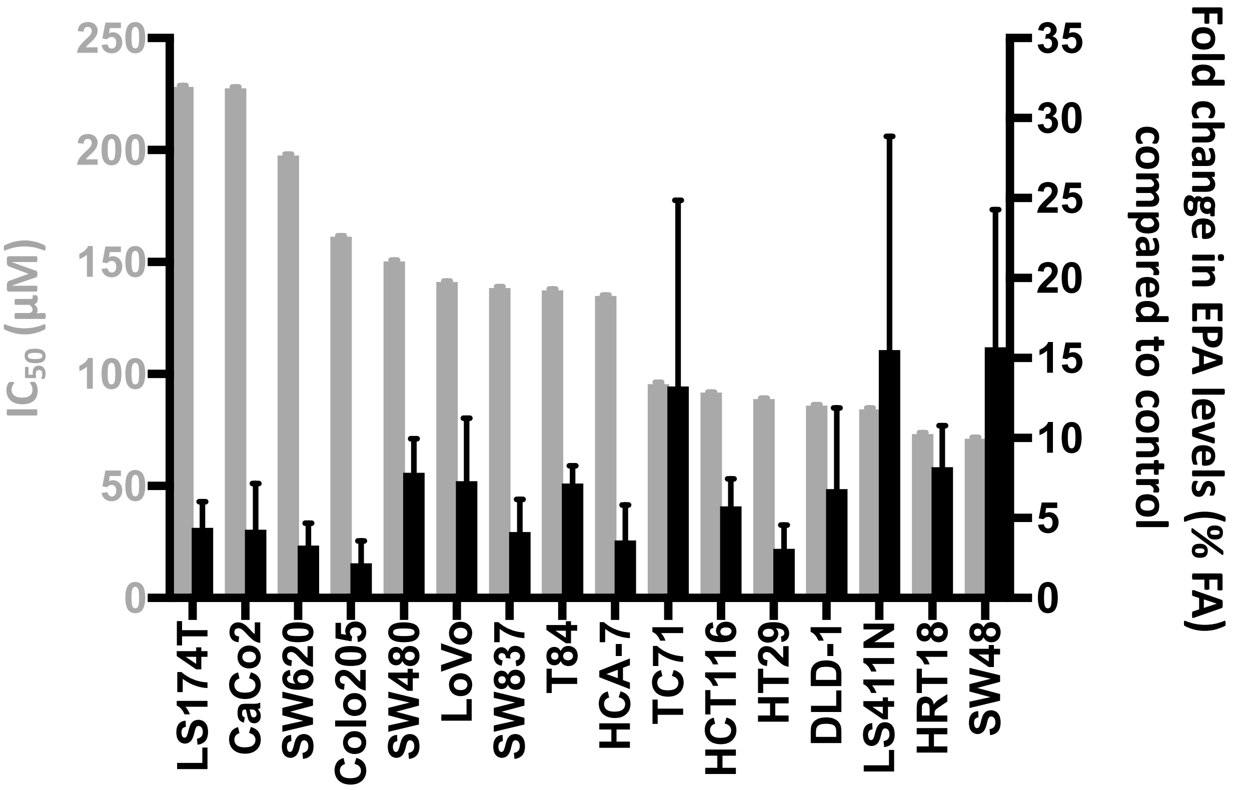

Supplementary fig. S5: Inverse relationship between EPA sensitivity and changes in EPA content in human CRC cell lines. EPA levels were quantified by LC-ESI-MS/MS following 24 hr exposure to 5 μM EPA-FFA, Pearson correlation r = -0.57, p = 0.02. Bars represents the mean fold change in EPA amount (ng/million cells) between EPA-treated and carrier-controls ± SEM for 2 independent experiments.

a b

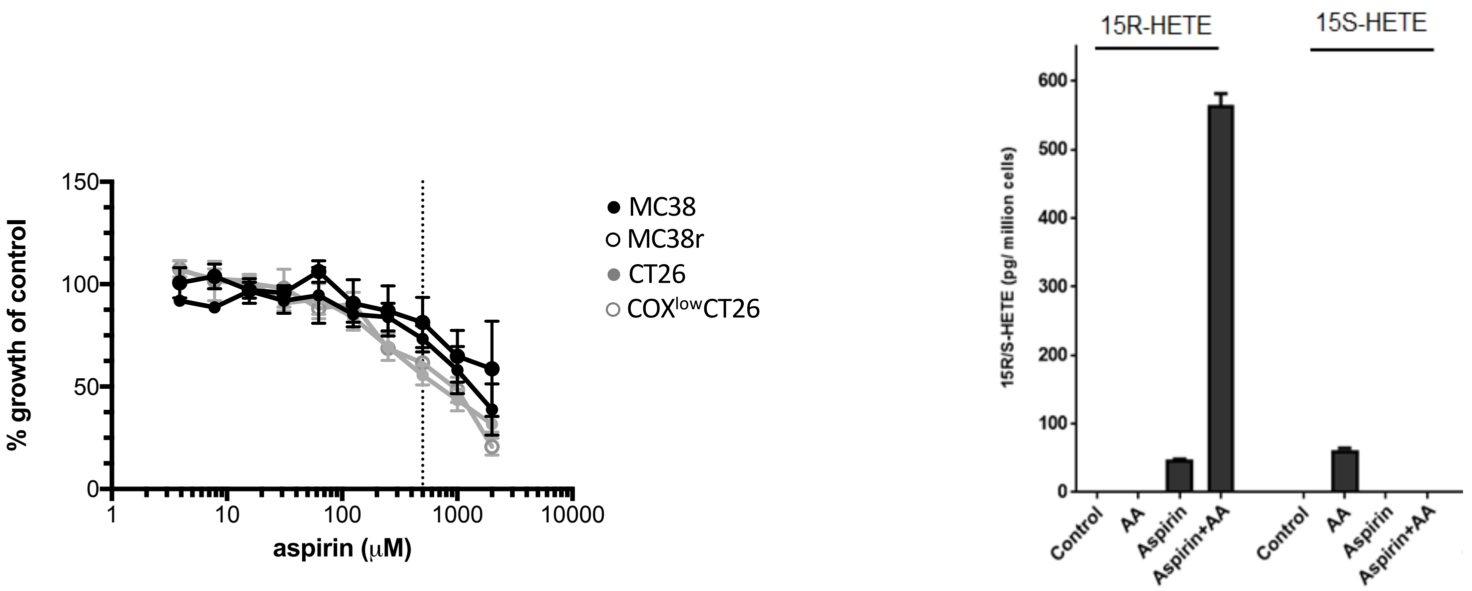

Supplementary fig. S6: Aspirin (500 µM) is pharmacologically active without inducing significant toxicity. a) Aspirin exposure (500 µM) did not induce cell death following continuous exposure in mouse MC38, MC38r, CT26 and COX^{low}CT26 CRC cells. Data are expressed as mean ± SEM for n=3 independent experiments. b) Aspirin (500 µM, 3hr) exposure induced a switch in chirality of 15-HETE metabolised from arachidonic acid (AA, 1 µM, 3h) from S to R form. Data are expressed as mean ± SEM for n=2 independent experiments

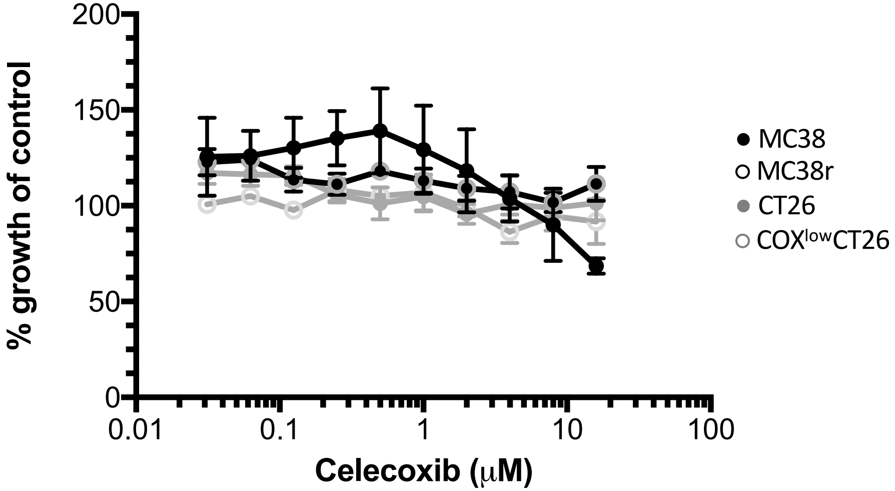

Supplementary fig. S7: Celecoxib (0.5 μM) does not induce toxicity following continuous exposure in mouse MC38, MC38r, CT26 and COX^{low}CT26 CRC cell lines. Data are expressed as the mean ± SEM percentage cell growth compared with control cells for n=3 independent experiments.

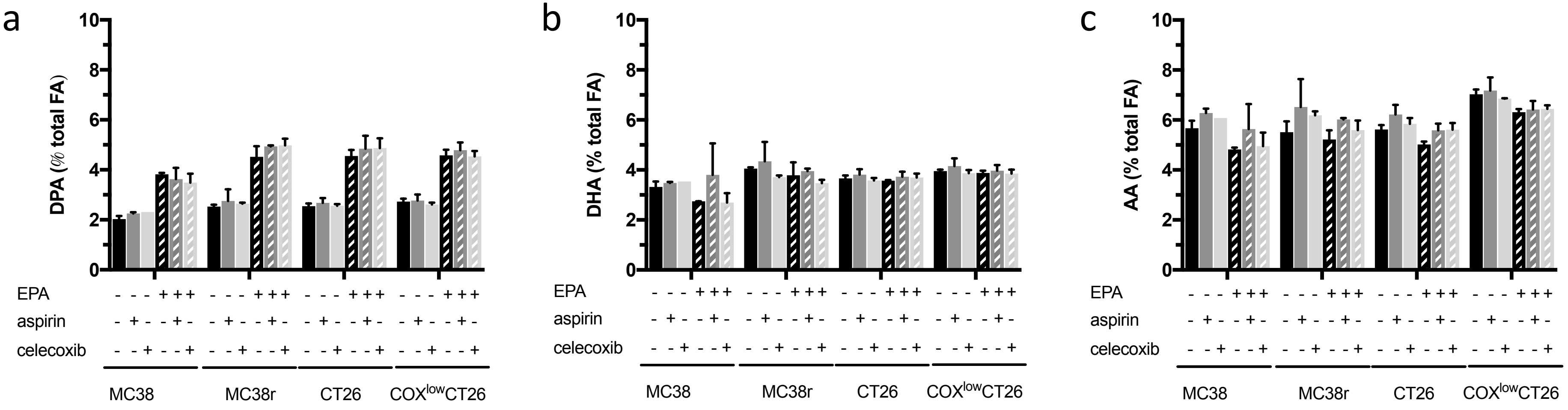

Supplementary fig. S8: Polyunsaturated fatty acid content of mouse CRC cell lines. DPA (a) content significantly increased in all cell samples treated with 5 µM EPA-FFA (T-test $p<0.01$). EPA-FFA exposure was not associated with a change in DHA (b) nor AA (c) content in these cell lines. Fatty acids were quantified by LC-ESI-MS/MS following 24 hr exposure to carrier only or 5 µM EPA-FFA in the presence or absence of 500 µM aspirin or 0.5 µM celecoxib. Data represent the mean ± SEM percentage of total fatty acid for DPA (a), DHA (b) and AA (c) of 3 independent experiments. In absence of error bar, the data represent n=1 as samples failed analysis.

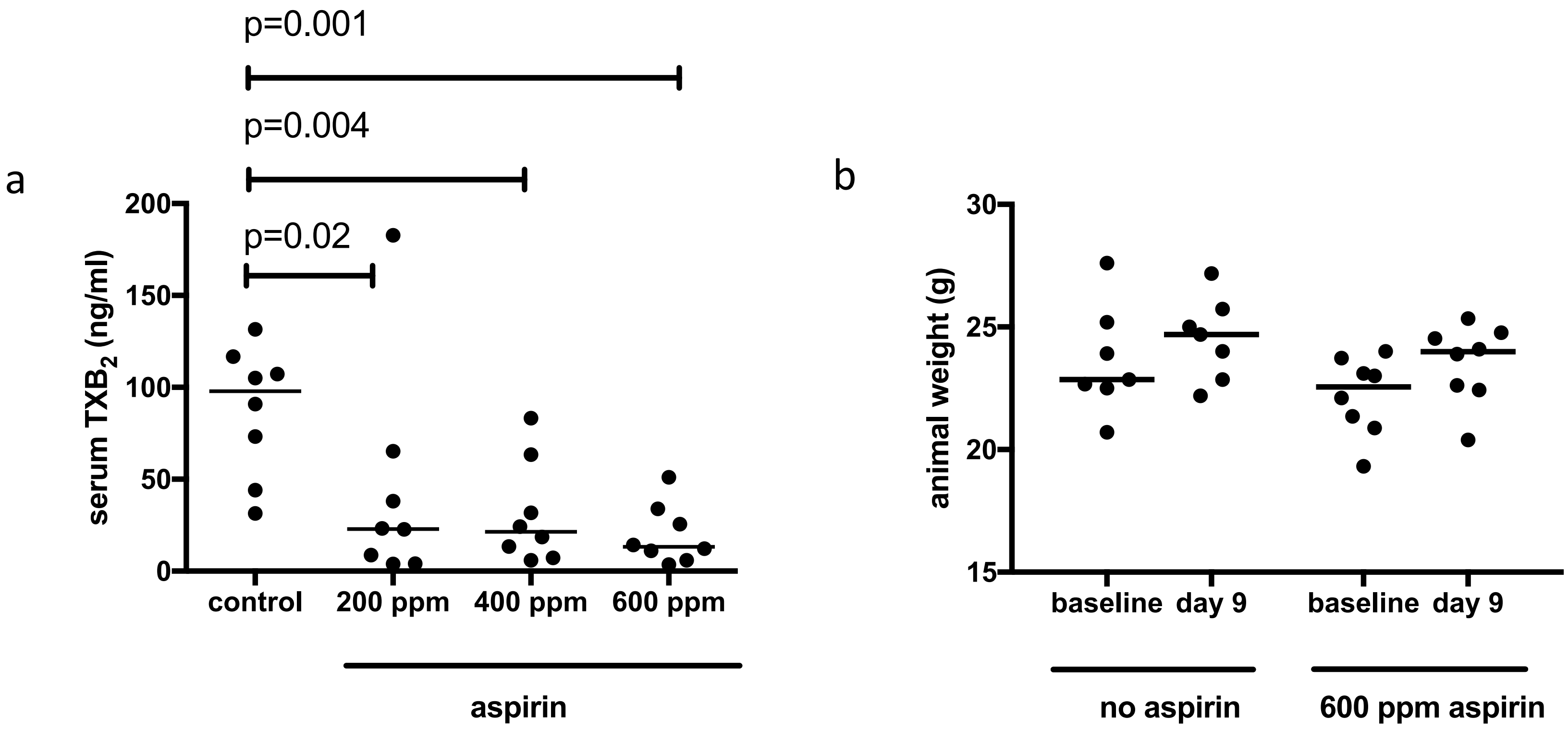

Supplementary fig. S9: Pharmacodynamic profile of aspirin in CD1-nude mice. CD1-nude mice (n=8 per group) were fed experimental diets containing a range of aspirin concentrations for 9 days. a) Serum TXB_2 level at sacrifice. b) Animal weight. The horizontal line within the box indicates the median value for each group. P values are for Mann-Whitney test.

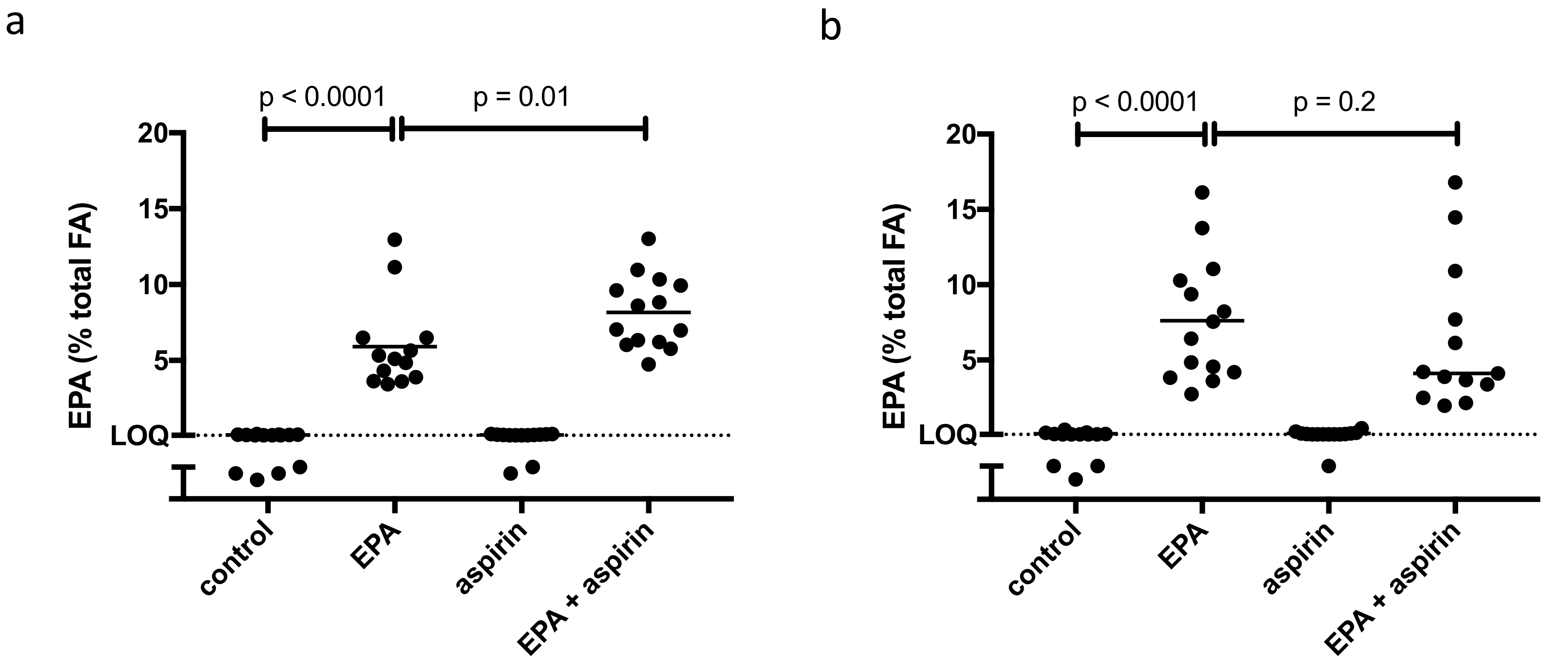

Supplementary fig. S10: EPA levels in mouse CRC cell tumour tissue. MC38 (a) and MC38R (b) mouse CRC cells were grown as subcutaneous tumours in CD1-nude mice fed experimental diet containing EPA, aspirin (600ppm) or a combination of EPA and aspirin (n=14 per group). The horizontal line indicates the median value for each group. The limit of quantification (LOQ) for EPA was 0.06% of total tissue FA content. P values are for Mann-Whitney test.

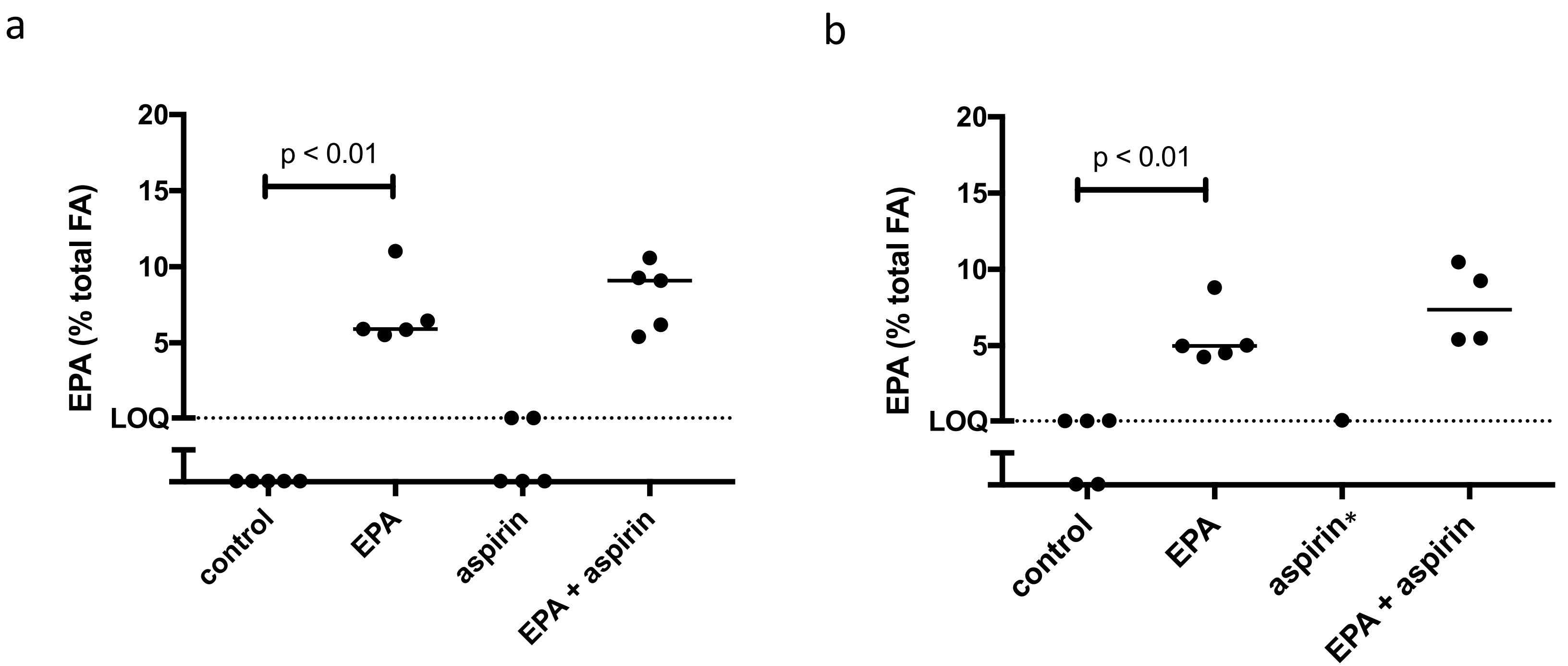

Supplementary fig. S11: EPA levels in human CRC cell tumour tissue. SW620 cells (a) and HCA-7 (b) human CRC cells were grown as subcutaneous tumours in CD1-nude mice fed experimental diet containing EPA, aspirin (600ppm) or a combination or EPA and aspirin (n=5 per group). The horizontal line indicates the median value for each group. The limit of quantification (LOQ) for EPA was 0.06% of total tissue fatty acids. P values are for the Mann-Whitney U test.
