# Supplementary material for: Cyclooxygenase activity mediates colorectal cancer cell resistance to the omega-3 polyunsaturated fatty acid eicosapentaenoic acid
Source: Cancer Chemother Pharmacol. 2020 Oct 11;87(2):173–84. doi: 10.1007/s00280-020-04157-2 (PMC7870614; doi:10.1007/s00280-020-04157-2)
Supplement: Supplementary file 4 — Supplementary file4 (PDF 90 kb) [file 280_2020_4157_MOESM4_ESM.pdf]

Cyclooxygenase activity mediates colorectal cancer cell resistance to the omega-3 polyunsaturated fatty acid eicosapentaenoic acid. Cancer Chemotherapy and Pharmacology.

Milene Volpato, Nicola Ingram, Sarah L Perry, Jade Spencer, Amanda D Race, Catriona Marshall, John M Hutchinson, Anna Nicolaou, Paul M Loadman, P Louise Coletta and Mark A Hull.

Corresponding author contact: Leeds Institute of Medical Research at St James's, University of Leeds, St James's University Hospital, Leeds LS9 7TF, United Kingdom, m.volpato@leeds.ac.uk

Table S3: Differential gene expression in MC38 and MC38r mouse CRC cells

Gene symbol	Gene name	Proposed function (IPA® analysis)	Fold-change in transcript level - EPA-treated MC38 cells compared with untreated MC38 cells (array 1)	Fold-change in transcript level - MC38r cells compared with MC38 cells (array 2)
Lox	Lysyl oxidase	collagen cross-linking	+2.4	-185.3
Col6a1	Collagen	extracellular matrix	+2.1	-7.0
P4ha1	Prolyl 4-hydroxylase α1	folding of collagen chain	+2.1	-2.6
Mrgprf	Mas-related G-coupled protein receptor F	orphan GPCR	+2.3	-3.5
Angptl6	Angiopoietin-like 6	chemo-attraction and inflammation	-2.6	+18.8
Ptgs1	Cyclooxygenase-1	prostaglandin synthesis	No significant change	+3.2

+, increase; -, decrease
